# The genome sequence of a heart cockle, *Fragum fragum *(Linnaeus, 1758)

**DOI:** 10.12688/wellcomeopenres.21134.1

**Published:** 2024-03-07

**Authors:** Ruiqi Li, Jingchun Li, Sarah Lemer, Jose Victor Lopez, Graeme Oatley, Isabelle Ailish Clayton-Lucey, Elizabeth Sinclair, Eerik Aunin, Noah Gettle, Camilla Santos, Michael Paulini, Haoyu Niu, Victoria McKenna, Rebecca O’Brien, Wellcome Sanger Institute Tree of Life Management, Samples and Laboratory Team, Wellcome Sanger Institute Scientific Operations: Sequencing Operations, Wellcome Sanger Institute Tree of Life Core Informatics Team, EBI Aquatic Symbiosis Genomics Data Portal Team, Aquatic Symbiosis Genomics Project Leadership

**Affiliations:** 1 Ecology & Evolutionary Biology, University of Colorado Boulder, Boulder, Colorado, USA; 2 Museum of Natural History, University of Colorado Boulder, Boulder, Colorado, USA; 3 University of Guam Marine Lab, Mangilao, Guam; 4 Centre for Molecular Biodiversity Research, Leibniz Institute for the Analysis of Biodiversity Change, Hamburg, Germany; 5 Department of Biological Sciences, Nova Southeastern University, Dania Beach, Florida, USA; 6 Tree of Life, Wellcome Sanger Institute, Hinxton, England, UK

**Keywords:** Fragum fragum, heart cockle, genome sequence, chromosomal, Veneroida

## Abstract

We present a genome assembly from an individual specimen of *Fragum fragum* (a heart cockle; Mollusca; Bivalvia; Veneroida; Cardiidae). The genome sequence is 1,153.1 megabases in span. Most of the assembly is scaffolded into 19 chromosomal pseudomolecules. The mitochondrial genome has also been assembled and is 22.36 kilobases in length. Gene annotation of this assembly on Ensembl identified 17,262 protein coding genes.

## Species taxonomy

Eukaryota; Opisthokonta; Metazoa; Eumetazoa; Bilateria; Protostomia; Spiralia; Lophotrochozoa; Mollusca; Bivalvia; Autobranchia; Heteroconchia; Euheterodonta; Imparidentia; Neoheterodontei; Cardiida; Cardioidea; Cardiidae; Fraginae; *Fragum*; *Fragum fragum* Linnaeus, 1758 (NCBI:txid80821).

## Background

The marine bivalve subfamily Fraginae (heart cockles) includes more than 50 described species found in temperate and tropical waters worldwide ( Kirkendale, 2009). It contains one non-symbiotic clade and one symbiotic clade ( Li *et al.*, 2020), in which species maintain a photosymbiotic relationship with dinoflagellate symbionts belonging to the family Symbiodiniaceae ( Kirkendale & Paulay, 2017). This provides a perfect opportunity to conduct comparative studies to reveal the origin and molecular mechanism of animal photosymbiosis. *Fragum fragum* is one of the most broadly distributed species in its genus, ranging from the eastern African coast to the Tuamotu Archipelago ( Kirkendale *et al.*, 2021). It is semi-epifaunal and dwells in sandy sediments. It is known to be associated with symbionts from the genus *Cladocopium* ( Li *et al.*, 2018). *F. fragum* shells exhibit unique morphological adaptations to photosymbiosis, including a large surface-to-volume ratio, flattened posterior side, and the presence of transparent shell microstructures (windows) ( Kirkendale, 2009). Studying the whole genome assembly of *F. fragum* has the potential to address important questions regarding the molecular mechanism, evolution, and adaptation of photosymbiosis. For example, what genetic innovations allow them to host symbionts in a specialised tubular system? What is the molecular mechanism underlying the shell window structure, which allows them to meet symbionts’ light requirement? Does *F. fragum* share similar molecular mechanisms of photosymbiosis with other photosymbiotic organisms, such as corals? This high-quality genome lays the foundation for creating a new model system to investigate photosymbiosis in Metazoa.

## Genome sequence report

**Figure 1.**
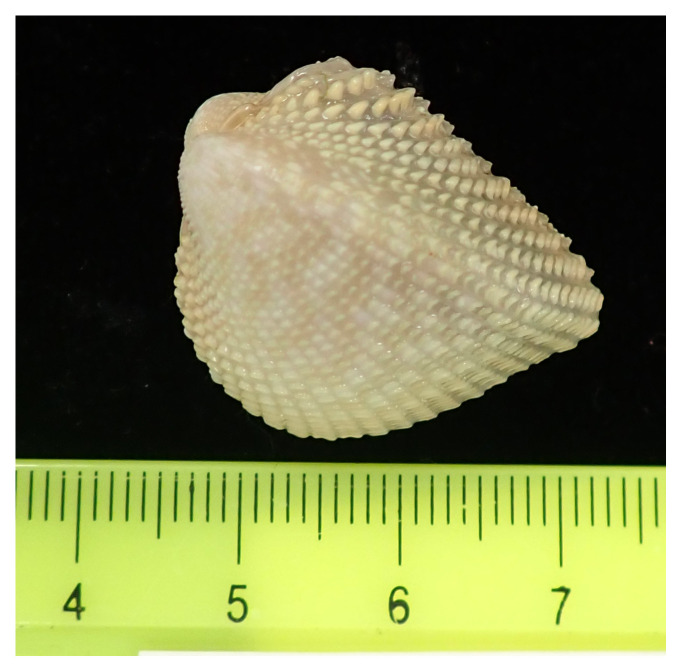
Photograph the *Fragum fragum* specimen (specimen ID NSU0013502, ToLID xbFraFrag2) used for genome sequencing.

The genome was sequenced from a specimen of *Fragum fragum* ( Figure 1) collected from East Hagatna Bay Beach, Guam, USA (13.49, 144.77). A total of 14-fold coverage in Pacific Biosciences single-molecule HiFi long reads was generated. Primary assembly contigs were scaffolded with chromosome conformation Hi-C data. Manual assembly curation corrected 98 missing joins or mis-joins and removed 99 haplotypic duplications, reducing the assembly length by 4.02% and the scaffold number by 68.80%, and increasing the scaffold N50 by 0.31%.

**Figure 2.**
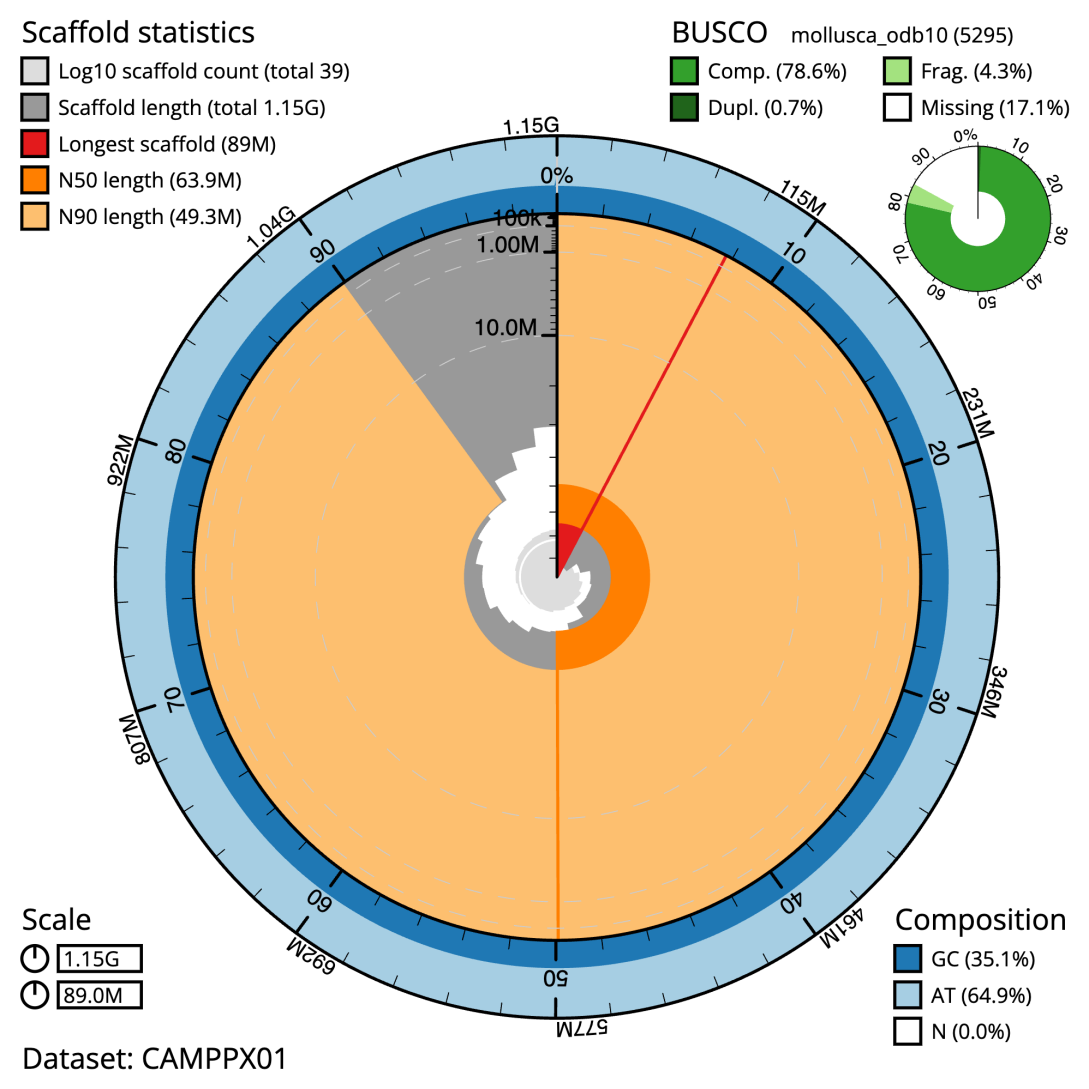
Genome assembly of *Fragum fragum*, xbFraFrag2.1: metrics. The BlobToolKit Snailplot shows N50 metrics and BUSCO gene completeness. The main plot is divided into 1,000 size-ordered bins around the circumference with each bin representing 0.1% of the 1,153,092,446 bp assembly. The distribution of scaffold lengths is shown in dark grey with the plot radius scaled to the longest scaffold present in the assembly (88,961,846 bp, shown in red). Orange and pale-orange arcs show the N50 and N90 scaffold lengths (63,927,008 and 49,258,289 bp), respectively. The pale grey spiral shows the cumulative scaffold count on a log scale with white scale lines showing successive orders of magnitude. The blue and pale-blue area around the outside of the plot shows the distribution of GC, AT and N percentages in the same bins as the inner plot. A summary of complete, fragmented, duplicated and missing BUSCO genes in the mollusca_odb10 set is shown in the top right. An interactive version of this figure is available at https://blobtoolkit.genomehubs.org/view/CAMPPX01/dataset/CAMPPX01/snail.

**Figure 3.**
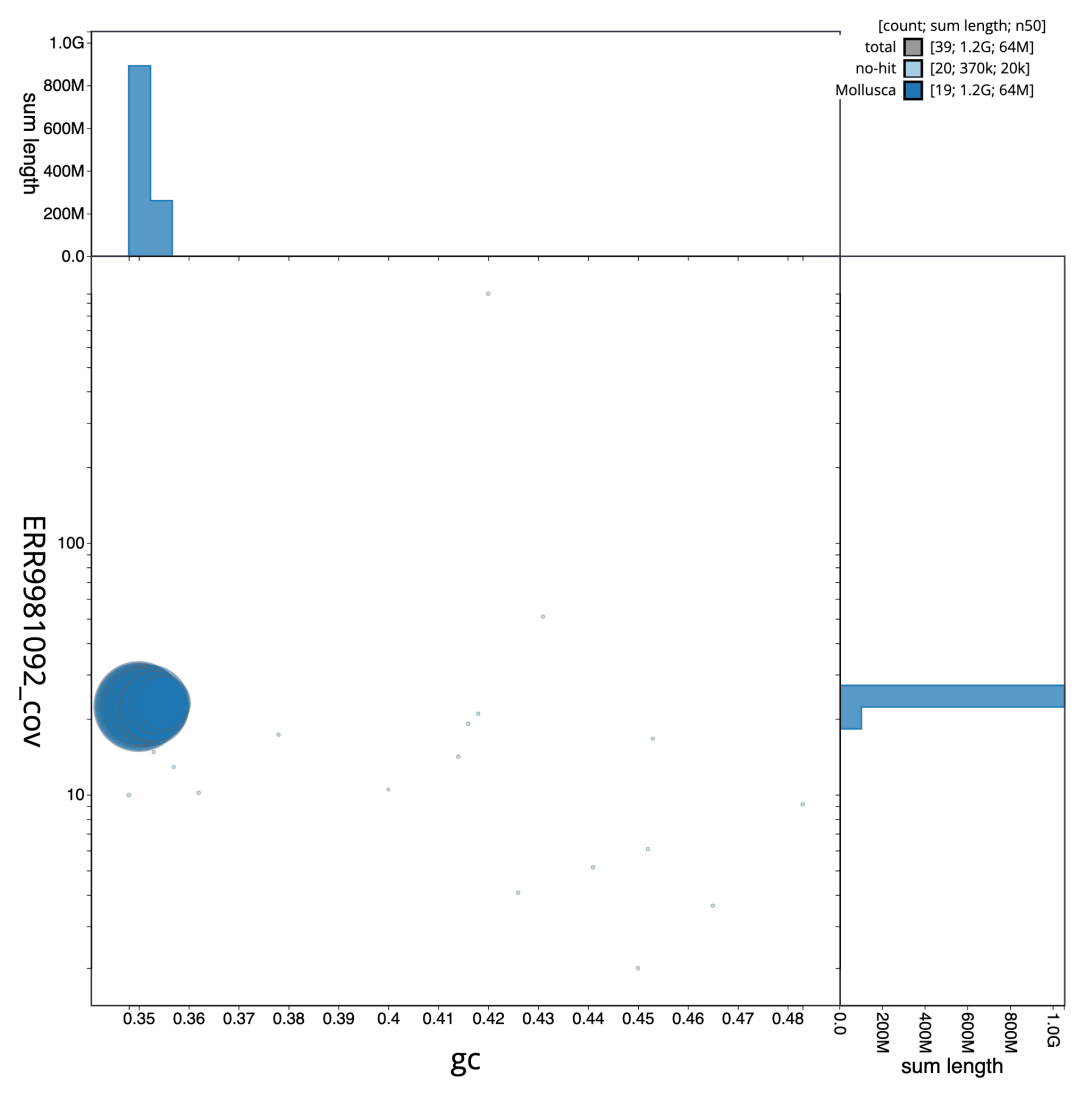
Genome assembly of *Fragum fragum*, xbFraFrag2.1: BlobToolKit GC-coverage plot. Scaffolds are coloured by phylum. Circles are sized in proportion to scaffold length. Histograms show the distribution of scaffold length sum along each axis. An interactive version of this figure is available at https://blobtoolkit.genomehubs.org/view/CAMPPX01/dataset/CAMPPX01/blob.

**Figure 4.**
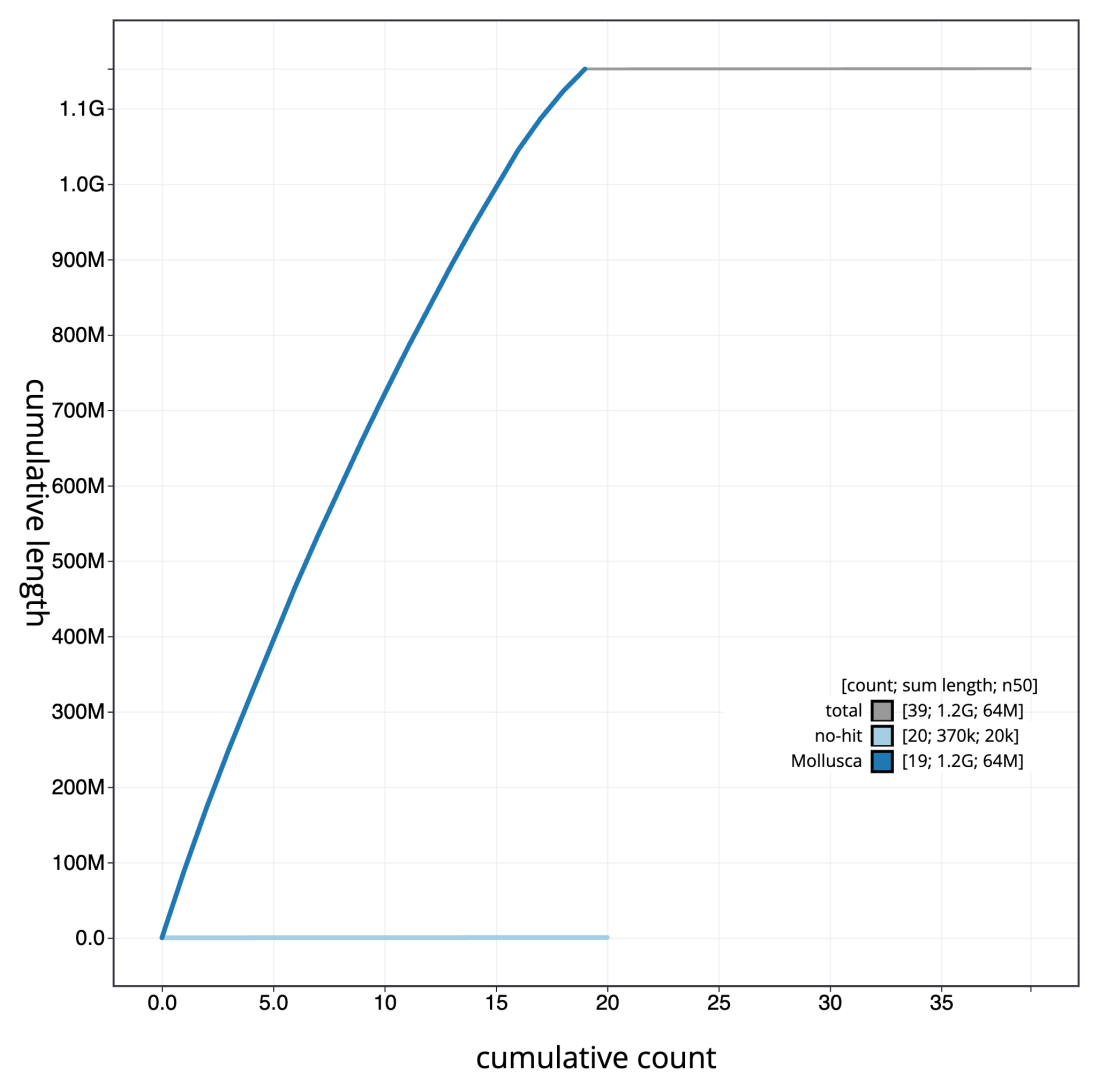
Genome assembly of *Fragum fragum*, xbFraFrag2.1: BlobToolKit cumulative sequence plot. The grey line shows cumulative length for all scaffolds. Coloured lines show cumulative lengths of scaffolds assigned to each phylum using the buscogenes taxrule. An interactive version of this figure is available at https://blobtoolkit.genomehubs.org/view/CAMPPX01/dataset/CAMPPX01/cumulative.

**Figure 5.**
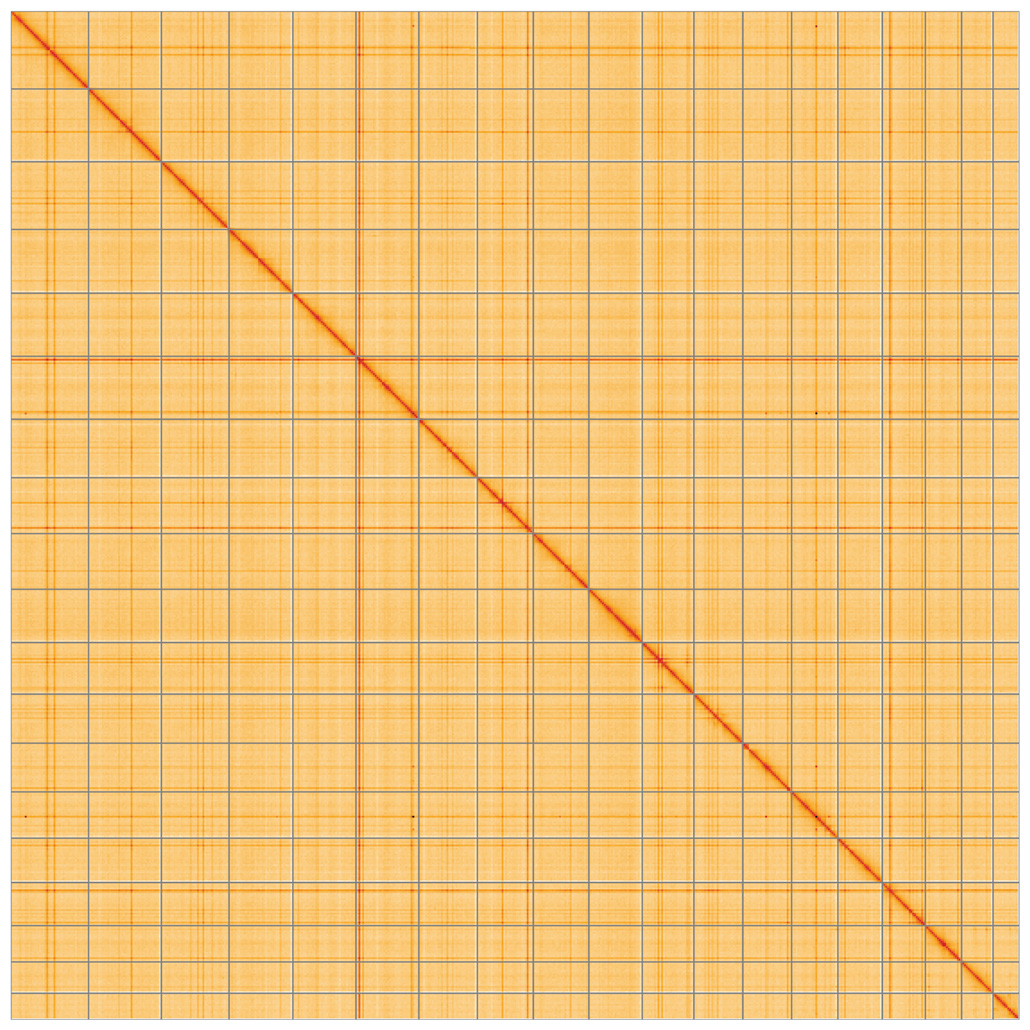
Genome assembly of *Fragum fragum*, xbFraFrag2.1: Hi-C contact map of the xbFraFrag2.1 assembly, visualised using HiGlass. Chromosomes are shown in order of size from left to right and top to bottom. An interactive version of this figure may be viewed at https://genome-note-higlass.tol.sanger.ac.uk/l/?d=DacnP7w_QK2J95ibXtLPgA.

The final assembly has a total length of 1153.1 Mb in 38 sequence scaffolds with a scaffold N50 of 63.9 Mb ( Table 1). The snail plot in Figure 2 provides a summary of the assembly statistics, while the distribution of assembly scaffolds on GC proportion and coverage is shown in Figure 3. The cumulative assembly plot in Figure 4 shows curves for subsets of scaffolds assigned to different phyla. Most (99.96%) of the assembly sequence was assigned to 19 chromosomal-level scaffolds. Chromosome-scale scaffolds confirmed by the Hi-C data are named in order of size ( Figure 5; Table 2). While not fully phased, the assembly deposited is of one haplotype. Contigs corresponding to the second haplotype have also been deposited. The mitochondrial genome was also assembled and can be found as a contig within the multifasta file of the genome submission.

**Table 1.** Genome data for *Fragum fragum*, xbFraFrag2.1.

Project accession data		
Assembly identifier	xbFraFrag2.1	
Species	*Fragum fragum*	
Specimen	xbFraFrag2	
NCBI taxonomy ID	80821	
BioProject	PRJEB54799	
BioSample ID	SAMEA9449766	
Isolate information	xbFraFrag2 (DNA sequencing) xbFraFrag1 (Hi-C and RNA sequencing)	
Assembly metrics *		*Benchmark*
Consensus quality (QV)	63.8	≥ *50*
*k*-mer completeness	100.0%	≥ *95%*
BUSCO **	C:78.6%[S:78.0%,D:0.7%], F:4.3%,M:17.1%,n:5,295	*C* ≥ *95%*
Percentage of assembly mapped to chromosomes	99.96%	≥ *95%*
Sex chromosomes	None	*localised homologous pairs*
Organelles	Mitochondrial genome: 22.36 kb	*complete single alleles*
Raw data accessions		
PacificBiosciences SEQUEL II	ERR9981092, ERR9981098	
Hi-C Illumina	ERR9988134	
PolyA RNA-Seq Illumina	ERR12245519	
Genome assembly		
Assembly accession	GCA_946902895.1	
*Accession of alternate haplotype*	GCA_946902885.1	
Span (Mb)	1153.1	
Number of contigs	883	
Contig N50 length (Mb)	2.3	
Number of scaffolds	38	
Scaffold N50 length (Mb)	63.9	
Longest scaffold (Mb)	88.96	
Genome annotation		
Number of protein-coding genes	17,262	
Number of non-coding genes	29,569	
Number of gene transcripts	55,288	

* Assembly metric benchmarks are adapted from column VGP-2020 of “Table 1: Proposed standards and metrics for defining genome assembly quality” from Rhie *et al.* (2021). ** BUSCO scores based on the mollusca_odb10 BUSCO set using version 5.3.2. C = complete [S = single copy, D = duplicated], F = fragmented, M = missing, n = number of orthologues in comparison. A full set of BUSCO scores is available at https://blobtoolkit.genomehubs.org/view/CAMPPX01/dataset/CAMPPX01/busco.

**Table 2.** Chromosomal pseudomolecules in the genome assembly of *Fragum fragum*, xbFraFrag2.

INSDC accession	Chromosome	Length (Mb)	GC%
OX336365.1	1	88.96	35.0
OX336366.1	2	83.18	35.0
OX336367.1	3	77.37	35.0
OX336368.1	4	72.9	35.0
OX336369.1	5	72.13	35.0
OX336370.1	6	71.9	35.0
OX336371.1	7	66.83	35.0
OX336372.1	8	63.93	35.0
OX336373.1	9	63.51	35.0
OX336374.1	10	60.88	35.0
OX336375.1	11	59.03	35.0
OX336376.1	12	56.02	35.0
OX336377.1	13	55.71	35.0
OX336378.1	14	52.86	35.5
OX336379.1	15	50.69	35.5
OX336380.1	16	49.26	35.5
OX336381.1	17	41.5	35.5
OX336382.1	18	35.8	35.5
OX336383.1	19	30.27	35.5
OX336384.1	MT	0.02	42.0

The estimated Quality Value (QV) of the final assembly is 63.8 with *k*-mer completeness of 100.0%, and the assembly has a BUSCO v5.3.2 completeness of 78.6% (single = 78.0%, duplicated = 0.7%), using the mollusca_odb10 reference set ( *n* = 5,295).

Metadata for specimens, barcode results, spectra estimates, sequencing runs, contaminants and pre-curation assembly statistics are given at https://links.tol.sanger.ac.uk/species/80821.

## Genome annotation report

The *Fragum fragum* genome (GCA_946902895.1) was annotated using the Ensembl rapid annotation pipeline at the European Bioinformatics Institute (EBI). The resulting annotation includes 55,288 transcribed mRNAs from 17,262 protein-coding and 29,262 non-coding genes ( Table 1; https://rapid.ensembl.org/Fragum_fragum_GCA_946902895.1/Info/Index).

## Methods

### Sample acquisition and nucleic acid extraction

The *Fragum fragum* specimens used for DNA sequencing (specimen ID NSU0013502, ToLID xbFraFrag2) and Hi-C and RNA sequencing (specimen ID NSU0013501, ToLID xbFraFrag1) were collected from East Hagatna Bay in front of Alupang Island, Guam, USA (latitude 13.49, longitude 144.77) on 2021-06-17, through a process of sifting sand with 1 mm metal sifters while snorkelling. The specimens were collected by Sarah Lemer (University of Guam Marine Lab) and identified by Ruiqi Li (University of Colorado, Boulder) and preserved by flash-freezing in liquid nitrogen.

The workflow for high molecular weight (HMW) DNA extraction at the Wellcome Sanger Institute (WSI) includes a sequence of core procedures: sample preparation; sample homogenisation, DNA extraction, fragmentation, and clean-up. In sample preparation, the xbFraFrag2 sample was weighed and dissected on dry ice ( Jay *et al.*, 2023). For sample homogenisation, muscle tissue was cryogenically disrupted using the Covaris cryoPREP ^®^ Automated Dry Pulverizer ( Narváez-Gómez *et al.*, 2023). HMW DNA was extracted using the Manual MagAttract v1 protocol ( Strickland *et al.*, 2023b). DNA was sheared into an average fragment size of 12–20 kb in a Megaruptor 3 system with speed setting 30 ( Todorovic *et al.*, 2023). Sheared DNA was purified by solid-phase reversible immobilisation ( Strickland *et al.*, 2023a): in brief, the method employs a 1.8X ratio of AMPure PB beads to sample to eliminate shorter fragments and concentrate the DNA. The concentration of the sheared and purified DNA was assessed using a Nanodrop spectrophotometer and Qubit Fluorometer and Qubit dsDNA High Sensitivity Assay kit. Fragment size distribution was evaluated by running the sample on the FemtoPulse system.

RNA was extracted from tissue of xbFraFrag1 in the Tree of Life Laboratory at the WSI using the RNA Extraction: Automated MagMax™ *mir*Vana protocol ( do Amaral *et al.*, 2023). The RNA concentration was assessed using a Nanodrop spectrophotometer and a Qubit Fluorometer using the Qubit RNA Broad-Range Assay kit. Analysis of the integrity of the RNA was done using the Agilent RNA 6000 Pico Kit and Eukaryotic Total RNA assay.

Protocols developed by the WSI Tree of Life laboratory are publicly available on protocols.io ( Denton *et al.*, 2023).

### Sequencing

Pacific Biosciences HiFi circular consensus DNA sequencing libraries were constructed according to the manufacturers’ instructions. Poly(A) RNA-Seq libraries were constructed using the NEB Ultra II RNA Library Prep kit. DNA and RNA sequencing was performed by the Scientific Operations core at the WSI on Pacific Biosciences SEQUEL II (HiFi) and Illumina NovaSeq 6000 (RNA-Seq) instruments. Hi-C data were also generated from muscle tissue of xbFraFrag1 using the Arima2 kit and sequenced on the Illumina NovaSeq 6000 instrument.

### Genome assembly, curation and evaluation

Assembly was carried out with Hifiasm ( Cheng *et al.*, 2021) and haplotypic duplication was identified and removed with purge_dups ( Guan *et al.*, 2020). The assembly was then scaffolded with Hi-C data ( Rao *et al.*, 2014) using YaHS ( Zhou *et al.*, 2023). The assembly was checked for contamination and corrected using the gEVAL system ( Chow *et al.*, 2016) as described previously ( Howe *et al.*, 2021). Manual curation was performed using gEVAL, HiGlass ( Kerpedjiev *et al.*, 2018) and Pretext ( Harry, 2022). The mitochondrial genome was assembled using MitoHiFi ( Uliano-Silva *et al.*, 2023), which runs MitoFinder ( Allio *et al.*, 2020) or MITOS ( Bernt *et al.*, 2013) and uses these annotations to select the final mitochondrial contig and to ensure the general quality of the sequence.

A Hi-C map for the final assembly was produced using bwa-mem2 ( Vasimuddin *et al.*, 2019) in the Cooler file format ( Abdennur & Mirny, 2020). To assess the assembly metrics, the *k*-mer completeness and QV consensus quality values were calculated in Merqury ( Rhie *et al.*, 2020). This work was done using Nextflow ( Di Tommaso *et al.*, 2017) DSL2 pipelines “sanger-tol/readmapping” ( Surana *et al.*, 2023a) and “sanger-tol/genomenote” ( Surana *et al.*, 2023b). The genome was analysed within the BlobToolKit environment ( Challis *et al.*, 2020) and BUSCO scores ( Manni *et al.*, 2021; Simão *et al.*, 2015) were calculated.

Table 3 contains a list of relevant software tool versions and sources.

**Table 3.** Software tools: versions and sources.

Software tool	Version	Source
BlobToolKit	4.2.1	https://github.com/blobtoolkit/blobtoolkit
BUSCO	5.3.2	https://gitlab.com/ezlab/busco
gEVAL	N/A	https://geval.org.uk/
Hifiasm	0.16.1-r375	https://github.com/chhylp123/hifiasm
HiGlass	1.11.6	https://github.com/higlass/higlass
Merqury	MerquryFK	https://github.com/thegenemyers/MERQURY.FK
MitoHiFi	2	https://github.com/marcelauliano/MitoHiFi
PretextView	0.2	https://github.com/wtsi-hpag/PretextView
purge_dups	1.2.3	https://github.com/dfguan/purge_dups
sanger-tol/genomenote	v1.0	https://github.com/sanger-tol/genomenote
sanger-tol/readmapping	1.1.0	https://github.com/sanger-tol/readmapping/tree/1.1.0
YaHS	yahs-1.1.91eebc2	https://github.com/c-zhou/yahs

### Genome annotation

The Ensembl Genebuild annotation system at the EBI ( Aken *et al.*, 2016) was used to generate annotation for the *Fragum fragum* assembly (GCA_946902895.1). Annotation was created primarily through alignment of transcriptomic data to the genome, with gap filling via protein-to-genome alignments of a select set of proteins from UniProt ( UniProt Consortium, 2019).

### Wellcome Sanger Institute – Legal and Governance

The materials that have contributed to this genome note have been supplied by a Tree of Life collaborator. The Wellcome Sanger Institute employs a process whereby due diligence is carried out proportionate to the nature of the materials themselves, and the circumstances under which they have been/are to be collected and provided for use. The purpose of this is to address and mitigate any potential legal and/or ethical implications of receipt and use of the materials as part of the research project, and to ensure that in doing so we align with best practice wherever possible. The overarching areas of consideration are:

•      Ethical review of provenance and sourcing of the material

•      Legality of collection, transfer and use (national and international)

Each transfer of samples is undertaken according to a Research Collaboration Agreement or Material Transfer Agreement entered into by the Tree of Life collaborator, Genome Research Limited (operating as the Wellcome Sanger Institute) and in some circumstances other Tree of Life collaborators.

## Data Availability

European Nucleotide Archive: *Fragum fragum* (heart cockle). Accession number PRJEB54799; https://identifiers.org/ena.embl/PRJEB54799 ( Wellcome Sanger Institute, 2022). The genome sequence is released openly for reuse. The *Fragum fragum* genome sequencing initiative is part of the Aquatics Symbiosis Genomics (ASG) project ( PRJEB43743). All raw sequence data and the assembly have been deposited in INSDC databases. Raw data and assembly accession identifiers are reported in Table 1.

## Author information

Members of the Wellcome Sanger Institute Tree of Life Management, Samples and Laboratory Team are listed here: https://doi.org/10.5281/zenodo.10066175.

Members of the Wellcome Sanger Institute Scientific Operations: Sequencing Operations are listed here: https://doi.org/10.5281/zenodo.10043364.

Members of the Wellcome Sanger Institute Tree of Life Core Informatics team are listed here: https://doi.org/10.5281/zenodo.10066637.

Members of the European Bioinformatics Institute ASG Data Portal team are listed here: https://doi.org//10.5281/zenodo.10076466.

Members of the Wellcome Sanger Institute/Aquatic Symbiosis Genomics Project Leadership are listed here: https://doi.org/10.5281/zenodo.10184833.

## References

[ref-1] Abdennur N Mirny LA : Cooler: Scalable storage for Hi-C data and other genomically labeled arrays. *Bioinformatics.* 2020;36(1):311–316. 10.1093/bioinformatics/btz540 31290943 PMC8205516

[ref-2] Aken BL Ayling S Barrell D : The Ensembl gene annotation system. *Database (Oxford).* 2016;2016: baw093. 10.1093/database/baw093 27337980 PMC4919035

[ref-3] Allio R Schomaker-Bastos A Romiguier J : MitoFinder: Efficient automated large-scale extraction of mitogenomic data in target enrichment phylogenomics. *Mol Ecol Resour.* 2020;20(4):892–905. 10.1111/1755-0998.13160 32243090 PMC7497042

[ref-4] Bernt M Donath A Jühling F : MITOS: Improved *de novo* metazoan mitochondrial genome annotation. *Mol Phylogenet Evol.* 2013;69(2):313–319. 10.1016/j.ympev.2012.08.023 22982435

[ref-5] Challis R Richards E Rajan J : BlobToolKit - Interactive Quality Assessment of Genome Assemblies. *G3 (Bethesda).* 2020;10(4):1361–1374. 10.1534/g3.119.400908 32071071 PMC7144090

[ref-6] Cheng H Concepcion GT Feng X : Haplotype-resolved *de novo* assembly using phased assembly graphs with hifiasm. *Nat Methods.* 2021;18(2):170–175. 10.1038/s41592-020-01056-5 33526886 PMC7961889

[ref-7] Chow W Brugger K Caccamo M : gEVAL - a web-based browser for evaluating genome assemblies. *Bioinformatics.* 2016;32(16):2508–2510. 10.1093/bioinformatics/btw159 27153597 PMC4978925

[ref-8] Denton A Yatsenko H Jay J : Sanger Tree of Life Wet Laboratory Protocol Collection V.1. *protocols.io.* 2023. 10.17504/protocols.io.8epv5xxy6g1b/v1

[ref-9] Di Tommaso P Chatzou M Floden EW : Nextflow enables reproducible computational workflows. *Nat Biotechnol.* 2017;35(4):316–319. 10.1038/nbt.3820 28398311

[ref-10] do Amaral RJV Bates A Denton A : Sanger Tree of Life RNA Extraction: Automated MagMax ^TM^ mirVana. *protocols.io.* 2023. 10.17504/protocols.io.6qpvr36n3vmk/v1

[ref-11] Guan D McCarthy SA Wood J : Identifying and removing haplotypic duplication in primary genome assemblies. *Bioinformatics.* 2020;36(9):2896–2898. 10.1093/bioinformatics/btaa025 31971576 PMC7203741

[ref-12] Harry E : PretextView (Paired REad TEXTure Viewer): A desktop application for viewing pretext contact maps. 2022; [Accessed 19 October 2022]. Reference Source

[ref-13] Howe K Chow W Collins J : Significantly improving the quality of genome assemblies through curation. *GigaScience.* Oxford University Press,2021;10(1): giaa153. 10.1093/gigascience/giaa153 33420778 PMC7794651

[ref-14] Jay J Yatsenko H Narváez-Gómez JP : Sanger Tree of Life Sample Preparation: Triage and Dissection. *protocols.io.* 2023. 10.17504/protocols.io.x54v9prmqg3e/v1

[ref-15] Kerpedjiev P Abdennur N Lekschas F : HiGlass: web-based visual exploration and analysis of genome interaction maps. *Genome Biol.* 2018;19(1): 125. 10.1186/s13059-018-1486-1 30143029 PMC6109259

[ref-16] Kirkendale L : Their Day in the Sun: molecular phylogenetics and origin of photosymbiosis in the ‘other’ group of photosymbiotic marine bivalves (Cardiidae: Fraginae). 2009;97(2):448–465. 10.1111/j.1095-8312.2009.01215.x

[ref-17] Kirkendale L Paulay G : Photosymbiosis in Bivalvia. *Treatise Online.* 2017;1(89). Reference Source

[ref-18] Kirkendale L ter Poorten JJ Middelfart P : A new photosymbiotic marine bivalve with window shell microstructure (Cardiidae: Fraginae) *Phuket Marine Biological Center Research Bulletin.* 2021;78:125–138. Reference Source

[ref-19] Li J Lemer S Kirkendale L : Shedding light: a phylotranscriptomic perspective illuminates the origin of photosymbiosis in marine bivalves. *BMC Evol Biol.* 2020;20(1):50. 10.1186/s12862-020-01614-7 32357841 PMC7195748

[ref-20] Li J Volsteadt M Kirkendale L : Characterizing Photosymbiosis Between Fraginae Bivalves and *Symbiodinium* Using Phylogenetics and Stable Isotopes. *Front Ecol Evol.* 2018;6:1–11. 10.3389/fevo.2018.00045

[ref-21] Manni M Berkeley MR Seppey M : BUSCO Update: Novel and Streamlined Workflows along with Broader and Deeper Phylogenetic Coverage for Scoring of Eukaryotic, Prokaryotic, and Viral Genomes. *Mol Biol Evol.* 2021;38(10):4647–4654. 10.1093/molbev/msab199 34320186 PMC8476166

[ref-22] Narváez-Gómez JP Mbye H Oatley G : Sanger Tree of Life Sample Homogenisation: Covaris cryoPREP® Automated Dry Pulverizer V.1. *protocols.io.* 2023. 10.17504/protocols.io.eq2lyjp5qlx9/v1

[ref-23] Rao SSP Huntley MH Durand NC : A 3D map of the human genome at kilobase resolution reveals principles of chromatin looping. *Cell.* 2014;159(7):1665–1680. 10.1016/j.cell.2014.11.021 25497547 PMC5635824

[ref-24] Rhie A McCarthy SA Fedrigo O : Towards complete and error-free genome assemblies of all vertebrate species. *Nature.* 2021;592(7856):737–746. 10.1038/s41586-021-03451-0 33911273 PMC8081667

[ref-25] Rhie A Walenz BP Koren S : Merqury: reference-free quality, completeness, and phasing assessment for genome assemblies. *Genome Biol.* 2020;21(1): 245. 10.1186/s13059-020-02134-9 32928274 PMC7488777

[ref-26] Simão FA Waterhouse RM Ioannidis P : BUSCO: assessing genome assembly and annotation completeness with single-copy orthologs. *Bioinformatics.* 2015;31(19):3210–3212. 10.1093/bioinformatics/btv351 26059717

[ref-27] Strickland M Cornwell C Howard C : Sanger Tree of Life Fragmented DNA clean up: Manual SPRI. *protocols.io.* 2023a. 10.17504/protocols.io.kxygx3y1dg8j/v1

[ref-28] Strickland M Moll R Cornwell C : Sanger Tree of Life HMW DNA Extraction: Manual MagAttract, *protocols.io.* 2023b. 10.17504/protocols.io.6qpvr33novmk/v1

[ref-29] Surana P Muffato M Qi G : sanger-tol/readmapping: sanger-tol/readmapping v1.1.0 - Hebridean Black (1.1.0). *Zenodo.* 2023a. 10.5281/zenodo.7755669

[ref-30] Surana P Muffato M Sadasivan Baby C : sanger-tol/genomenote (v1.0.dev). *Zenodo.* 2023b. 10.5281/zenodo.6785935

[ref-31] Todorovic M Sampaio F Howard C : Sanger Tree of Life HMW DNA Fragmentation: Diagenode Megaruptor®3 for PacBio HiFi. *protocols.io.* 2023. 10.17504/protocols.io.8epv5x2zjg1b/v1

[ref-32] Uliano-Silva M Ferreira JGRN Krasheninnikova K : MitoHiFi: a python pipeline for mitochondrial genome assembly from PacBio high fidelity reads. *BMC Bioinformatics.* 2023;24(1): 288. 10.1186/s12859-023-05385-y 37464285 PMC10354987

[ref-33] UniProt Consortium: UniProt: a worldwide hub of protein knowledge. *Nucleic Acids Res.* 2019;47(D1):D506–D515. 10.1093/nar/gky1049 30395287 PMC6323992

[ref-34] Vasimuddin M Misra S Li H : Efficient Architecture-Aware Acceleration of BWA-MEM for Multicore Systems. In: *2019 IEEE International Parallel and Distributed Processing Symposium (IPDPS)*. IEEE,2019;314–324. 10.1109/IPDPS.2019.00041

[ref-36] Wellcome Sanger Institute: The genome sequence of a heart cockle, *Fragum fragum* (Linnaeus, 1758). European Nucleotide Archive. [dataset], accession number PRJEB54799,2022.

[ref-35] Zhou C McCarthy SA Durbin R : YaHS: yet another Hi-C scaffolding tool. *Bioinformatics.* 2023;39(1): btac808. 10.1093/bioinformatics/btac808 36525368 PMC9848053

