# Supplementary material for: The genome sequence of a heart cockle, Fragum fragum (Linnaeus, 1758)
Source: Wellcome Open Res. 2024 Mar 7;9:129. [Version 1] doi: 10.12688/wellcomeopenres.21134.1 (PMC11234083; doi:10.12688/wellcomeopenres.21134.1)
Supplement: Peer review history

# Reviewer report: Reviewer response for version 1

**DOI:** 10.21956/wellcomeopenres.23378.r87893

**Published:** 2024-08-01

**Authors:** Masa-Aki Yoshida

**Affiliations:** 1 Shimane University, Matsue, Japan

Li *et al*. present the genome of the bivalve *Fragum fragum*. The assembled genome could be a valuable genetic resource for their unique adaptations to photosymbiosis. However, as other reviewers have noted, a couple of concerns should be addressed prior to indexing.

 The low score for the BUSCO analysis is problematic; mollusca_odb10 may not be appropriate for this species, and the BUSCO analysis for the entire reference set of metazoan_odb should also be re-run with closely related species and included in the text.

 Another point is that the possibility of contamination has not been adequately verified. The possibility of contamination of the genome of Cladocopium symbionts using the *Fragum* scaffold should be verified and stated with a long read mapper such as minimap.

Are sufficient details of methods and materials provided to allow replication by others?

Yes

Is the rationale for creating the dataset(s) clearly described?

Partly

Are the datasets clearly presented in a useable and accessible format?

Yes

Are the protocols appropriate and is the work technically sound?

Partly

Reviewer Expertise:

Comparatice genomics, Evolutionary biology

I confirm that I have read this submission and believe that I have an appropriate level of expertise to confirm that it is of an acceptable scientific standard, however I have significant reservations, as outlined above.

# Reviewer report: Reviewer response for version 1

**DOI:** 10.21956/wellcomeopenres.23378.r87889

**Published:** 2024-07-09

**Authors:** Zhenmin Bao

**Affiliations:** 1 MOE Key Laboratory of Marine Genetics and Breeding, College of Marine Life Sciences, Ocean University of China, Qingdao, China

Li *et al. *present the genome sequencing of *Fragum fragum*, a marine bivalve notable for its photosymbiotic relationships. This work is a valuable contribution to marine biology and genomics, thus underscoring the genome's quality.

 Although the assembly has a high N50 of 63.9 Mb, the BUSCO completeness is only 78.6%, which is relatively low and might suggest issues with assembly or annotation completeness. Moreover, the coverage of HiFi reads is only 14-fold, falling short of the typical 30-fold standard for high-quality genomes. Furthermore, the manuscript lacks detailed information on Hi-C read coverage, and reported Hi-C interactions within each chromosome appear week. The authors are encouraged to re-evaluate the gene annotation and validate the chromosomal structure to enhance the credibility and utility of the assembly.

Are sufficient details of methods and materials provided to allow replication by others?

Partly

Is the rationale for creating the dataset(s) clearly described?

Partly

Are the datasets clearly presented in a useable and accessible format?

Yes

Are the protocols appropriate and is the work technically sound?

Partly

Reviewer Expertise:

Mollusc genetics and genomics

I confirm that I have read this submission and believe that I have an appropriate level of expertise to confirm that it is of an acceptable scientific standard, however I have significant reservations, as outlined above.

# Reviewer report: Reviewer response for version 1

**DOI:** 10.21956/wellcomeopenres.23378.r87891

**Published:** 2024-07-09

**Authors:** Charles Plessy

**Affiliations:** 1 Okinawa Institute of Science and Technology, Okinawa, Japan

This reports follows the Tree of Life project's standard pattern, which makes it easy to review. I examined the contact map and the assembly statistics and found no obvious defect.

 The BUSCO score is below 80%, which raises the possibility that the assembly is not complete. However, publicly available BUSCO scores for the Cardiida order all show a very similar result, which is reassuring.  This should be noted in the manuscript as not every reader will be able to find this information online ( https://blobtoolkit.genomehubs.org/view/Cardiida#Datasets).

Are sufficient details of methods and materials provided to allow replication by others?

Yes

Is the rationale for creating the dataset(s) clearly described?

Yes

Are the datasets clearly presented in a useable and accessible format?

Yes

Are the protocols appropriate and is the work technically sound?

Yes

Reviewer Expertise:

Genome comparison by alignment

I confirm that I have read this submission and believe that I have an appropriate level of expertise to confirm that it is of an acceptable scientific standard.

# Reviewer report: Reviewer response for version 1

**DOI:** 10.21956/wellcomeopenres.23378.r87887

**Published:** 2024-07-05

**Authors:** Taro Maeda

**Affiliations:** 1 Keio University, Keio, Japan

The paper by Li et al., reports on the high-quality genome of *Fragum fragum*. While the genomic data for bivalves has been rapidly increasing in recent years, it remains insufficient to fully represent the diversity of these organisms. This paper provides a crucial reference dataset in this field.

 The authors claim to have achieved chromosome-level assembly, but I am concerned about the low BUSCO score of 78%. Is it a general trend for bivalve genomes to have lower BUSCO scores, or is this specific to the genome of *Fragum*?

*F. fragum* is known for its symbiotic relationship with microalgae, and individuals collected from the wild are expected to contain these symbionts. However, the details on how contamination from these symbiotic organisms was removed are unclear. The authors mention using the gEVAL system, but there is no description of how this genome browser was utilized to exclude contamination, and this browser is not publicly available.

 The information provided in the paper will significantly contribute to future research on *Fragum* species. However, a discussion on the completeness of the genome and the issue of contamination, both frequent challenges in genomic studies, would enhance the reliability of the data presented.

Are sufficient details of methods and materials provided to allow replication by others?

No

Is the rationale for creating the dataset(s) clearly described?

Partly

Are the datasets clearly presented in a useable and accessible format?

Yes

Are the protocols appropriate and is the work technically sound?

Partly

Reviewer Expertise:

Malacology, symbiosis

I confirm that I have read this submission and believe that I have an appropriate level of expertise to confirm that it is of an acceptable scientific standard, however I have significant reservations, as outlined above.

# Reviewer report: Reviewer response for version 1

**DOI:** 10.21956/wellcomeopenres.23378.r87888

**Published:** 2024-07-05

**Authors:** Daniel Garcia-Souto

**Affiliations:** 1 University of Santiago de Compostela, Santiago de Compostela, Galicia, Spain

Li *et al*. present the first comprehensive description of the genome of the bivalve *Fragum fragum*. The assembled genome demonstrates high-quality continuity; however, its completeness appears to be somewhat limited, with a BUSCO completeness score of 78.6%. This is relatively low compared to similar genomes produced using the same methodology and falls below the desired standards. Perhaps authors should re-run BUSCO against the full metazoan dataset as well.

 Nonetheless, this genome represents a valuable addition to the databases for an underrepresented species and deserves recognition in this manuscript.

 An important aspect to highlight is that *Fragum fragum* belongs to a group prone to exhibiting transmissible tumors, making this genomic data particularly significant for future studies on disease mechanisms and prevention in bivalves.

 On the lower side, the photograph of the shell provided in this manuscript might be insufficient for purely taxonomic purposes, as it does not highlight the hinge, which is typically the most informative taxonomic character for bivalves. However, the specimen is stored, and its identification is accurate, allowing specialized taxonomists to review it on-site if necessary.

Are sufficient details of methods and materials provided to allow replication by others?

Yes

Is the rationale for creating the dataset(s) clearly described?

Yes

Are the datasets clearly presented in a useable and accessible format?

Yes

Are the protocols appropriate and is the work technically sound?

Yes

Reviewer Expertise:

Molecular cytogenetics, repetitive DNA, genomics, malacology

I confirm that I have read this submission and believe that I have an appropriate level of expertise to confirm that it is of an acceptable scientific standard.
